# Novel Diagnosis and Therapeutics Approaches in Retina Diseases

**DOI:** 10.3390/life14101218

**Published:** 2024-09-24

**Authors:** Maurizio Mete, Daniela Iacovello, Emilia Maggio

**Affiliations:** 1 Department of Ophthalmology, IRRCS Sacro Cuore-Don Calabria Hospital, 3024 Negrar, Italy; daniela.iacovello@sacrocuore.it (D.I.); emilia.maggio@sacrocuore.it (E.M.); 2 Ophthalmology Unit, Dipartimento Di Scienze Mediche e Chirurgiche, Alma Mater Studiorum University of Bologna, 40126 Bologna, Italy; 3 IRCCS Azienda Ospedaliero-Universitaria di Bologna, 40126 Bologna, Italy

We have warmly welcomed the opportunity to propose the Special Issue titled “Novel Diagnosis and Therapeutics Approaches in Retina Diseases”, and numerous authors have decided to submit their works to it. This has resulted in a collection of extremely interesting and innovative articles, which has allowed us editors to learn new things. The focus of the published articles has been deliberately very specialized in order to provide readers with truly specific and innovative information. The main novelties that emerged concerned vitreoretinal diagnostics and surgery, but interesting multidisciplinary studies have also been received that see the ophthalmologist as an increasingly relevant figure in managing patient health, even regarding severe systemic diseases.

Starting with diagnostics, we have learned that there are alternative techniques for studying choroidal nevi. In addition to retinography, infrared, and ultrasound, retro-mode scanning laser ophthalmoscopy has proven to be an effective and easy-to-execute non-invasive method, useful for both diagnosis and follow-up [1]. Adaptive optics, considered a technique useful only in research contexts, has been presented to us as a clinically usable tool for studying acute posterior syphilitic placoid chorioretinopathy; although based on a limited number of cases, the authors have demonstrated that this technique is likely on the verge of being used more frequently in our daily clinics [2]. Another interesting study has added OCT reflectivity of foveal cysts as a biomarker for early response to anti-VEGF treatment of diabetic macular edema. The subject of biomarkers represents a hot topic, given the importance of increasingly personalizing treatment regimens for retinal vascular diseases [3]. Intraoperative OCT is also gaining more ground, allowing for more precise and aware surgery. We have indeed read that the use of liquid perfluorocarbon as an adjunct in macular pucker surgery can lead to better anatomical outcomes. This finding, of course, needs to be confirmed on a larger scale, but it is interesting to note that imaging is finding a place not only in surgical indication and follow-up but also during surgery to detail the dynamics following the surgeon’s actions [4].

An interesting contribution related to the clinical applications of artificial intelligence models could not be missed. Deep learning algorithms have indeed been used to predict the response to anti-VEGF therapy in patients with neovascular AMD [5]. This is an extremely promising approach that, stepping outside the experience of the individual physician, can allow for increasingly greater precision in optimizing treatment protocols, with favorable repercussions on the economic and healthcare front.

Numerous surgical relevance studies have also been presented. An innovative use of the amniotic membrane has been suggested, placed in the subretinal space in patients with severe AMD; the authors describe a partial restoration of the outer retinal layers. This finding needs to be confirmed on a larger scale, but nonetheless represents a very interesting observation, especially since nothing can currently be done in such cases [6]. In the surgical field, several interesting case reports have also been presented [7,8], which describe effective solutions for addressing everyday situations or potentially serious complications. We deemed it appropriate to include case reports because they are well-documented and presented with a comparison to the literature; they are indeed useful tools for immediate comparison between the author and the reader, who can draw inspiration from them or reflect upon them.

We have also included a review that examines relaxing retinotomies for the treatment of recurring or persistent macular holes. In recent years, numerous techniques have been described to promote the closure of refractory macular holes [9]. The sensation is often that a closure is obtained on OCT without a significant benefit to the retinal layers and therefore to vision. It is thus useful to promote literature reviews in order to better understand what to do and whether it is worth doing something when a macular hole does not close.

Numerous interesting multidisciplinary works have also been presented. In recent years, ophthalmic imaging techniques have indeed been acquiring an increasingly significant role in general medicine, suggesting a greater role for the ophthalmologist within the teams responsible for managing a patient with severe systemic diseases, such as renal insufficiency in diabetic patients [10] or pulmonary hypertension [11]. Last but not least, we have also hosted an article on basic science. In particular, the role of microglia in the pathogenesis of oxygen-induced maculopathy has been investigated, leading to the suggestion of new and promising therapeutic targets for a condition that currently lacks a cure [12].

In conclusion, when we defined the framework for this Special Issue, the goal was to present as wide a range of innovations as possible in the field of retina disorder diagnosis and therapy. We can say we have succeeded in this intent and hope that this issue will be of real interest to readers. From our side, we must express our gratitude to the authors who have allowed us to collect high-profile scientific works.
